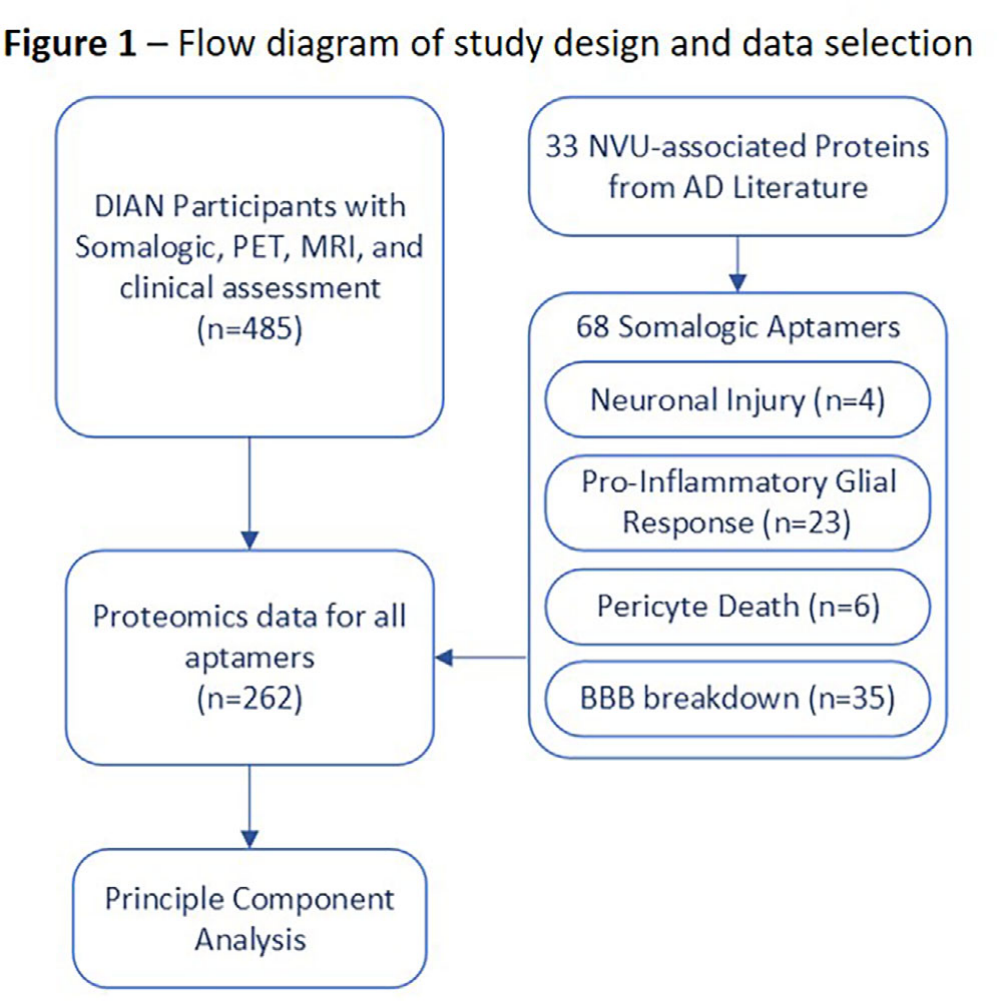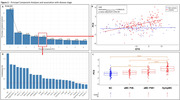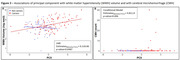# CSF proteomics related to the neurovascular unit are associated with white matter hyperintensity volumes and cerebral microhemorrhages in autosomal dominant Alzheimer disease

**DOI:** 10.1002/alz.093619

**Published:** 2025-01-09

**Authors:** Nelly Joseph‐Mathurin, Yuanyuan Shen, Karl A. Friedrichsen, Charles D. Chen, Austin A. McCullough, Jeremy F. Strain, Zahra Shirzadi, Gengsheng Chen, Parinaz Massoumzadeh, Brian A. Gordon, Jorge J. Llibre‐Guerra, Jason J. Hassenstab, Laura Ibanez, Ruijin Lu, Stephanie A. Schultz, Jasmeer P. Chhatwal, John C. Morris, Richard J. Perrin, Hongyu An, Chengjie Xiong, Robyn S. Klein, Clifford R. Jack, Randall J. Bateman, Eric McDade, Tammie L.S. Benzinger, Carlos Cruchaga

**Affiliations:** ^1^ Washington University in St. Louis, School of Medicine, St. Louis, MO USA; ^2^ Massachusetts General Hospital, Harvard Medical School, Boston, MA USA; ^3^ Washington University in St. Louis, St. Louis, MO USA; ^4^ Washington University in St. Louis School of Medicine, St. Louis, MO USA; ^5^ Mayo Clinic, Rochester, MN USA

## Abstract

**Background:**

Autosomal dominant Alzheimer disease (ADAD) is characterized by genetic mutations affecting the beta‐amyloid (Aß) pathway. However, vascular and immune factors play important roles which are not completely understood. Understanding the function of the neurovascular unit (NVU) comprised of neurons, glial cells, and vasculature, at different disease stages appears ideal to developing and evaluating therapeutics. Omics approaches can inform NVU changes and their disease associations.

**Methods:**

CSF proteomic data using the Somalogic® platform was generated from 485 participants in the DIAN study, who also had PiB‐PET and MRI assessing Aß burden, white matter hyperintensity (WMH), and cerebral microhemorrhage (CMH). From previously published AD studies, we identified 33 NVU‐associated proteins with corresponding Somalogic aptamers (Figure 1). We used Principal Component Analysis (PCA) on participants with complete data (n = 262, 166 mutation‐carriers and 96 non‐carriers) to find groups of proteins associated with mutation status and disease stage as measured with estimated year to symptom onset (EYO), PiB‐PET, and clinical status. Linear‐mixed effect models and two‐part zero‐inflated negative binomial mixed models further evaluated the link between significant principal components (PCs) and WMH and CMH, respectively. Models accounted for age, APOE‐e4 status, sex, and education.

**Results:**

The first ten PCs explained >60% of the variance in NVU‐related protein levels (Figure 2A) with only PC5 (4.8%) associated with mutation*EYO, suggesting relationship with ADAD‐specific disease progression (***p<0.0001, Figure 2B). While asymptomatic PiB‐negative individuals were not significantly different from non‐carriers, asymptomatic PiB‐positive individuals had higher PC5 scores than non‐carriers and symptomatic individuals scored still higher (**p<0.005, ***p<0.0001, Figure 2C). The main proteins composing PC5 were: neurofilament light‐chain, angiopoietin‐2, SMOC1, LRP1, E‐selectin, and sTREM2, suggesting strong association with proinflammatory glial response from neuronal injury (Figure 2D). PC5 was also associated with WMH volumes (*p<0.05, Figure 3A) and with CMH count in participants with CMH (**p<0.005, Figure 3B).

**Conclusions:**

This PCA approach indicates that some groups of NVU‐associated proteins are related with mutation status and disease stage, and that they are differentially associated with features of ADAD such as white matter integrity as measured by WMH volume. Weighted Correlation Network Analysis will be of interest to further understand these associations. Funding: K01AG080123, RF1‐AG044546, UF1AG032438